# Tetra­bromido(di-2-pyridyl­amine-κ^2^ *N* ^2^,*N* ^2′^)platinum(IV)

**DOI:** 10.1107/S160053681203379X

**Published:** 2012-08-01

**Authors:** Kwang Ha

**Affiliations:** a School of Applied Chemical Engineering, The Research Institute of Catalysis, Chonnam National University, Gwangju 500-757, Republic of Korea

## Abstract

The Pt^IV^ ion in the title complex, [PtBr_4_(C_10_H_9_N_3_)], is six-coordinated in a slightly distorted octa­hedral environment by two pyridine N atoms from a chelating di-2-pyridyl­amine (dpa) ligand and four Br^−^ anions. The complex mol­ecule has mirror symmetry, with the Pt^IV^ atom, two Br atoms and the central N atom of the dpa ligand lying on the mirror plane. The dpa ligand is not planar, showing a dihedral angle of 34.7 (2)° between the pyridine rings. The complex mol­ecules are connected by inter­molecular N—H⋯Br hydrogen bonds, forming chains along [001]. Inter­molecular C—H⋯Br hydrogen bonds and π–π inter­actions between the pyridine rings [centroid–centroid distance = 3.667 (4) Å] are also observed.

## Related literature

For the structures of the related complexes [PtCl_4_(dpa)] and [PtBr_2_(dpa)], see: Ha (2011 ▶, 2012 ▶).
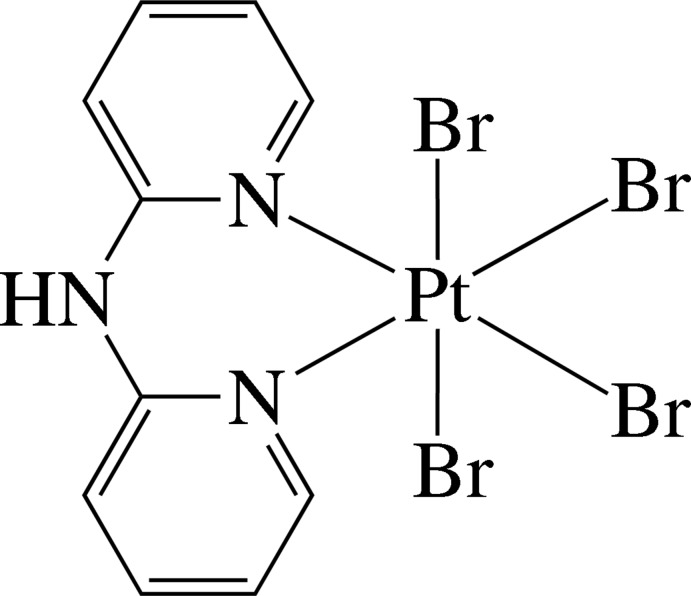


## Experimental

### 

#### Crystal data

[PtBr_4_(C_10_H_9_N_3_)]
*M* *_r_* = 685.93
Monoclinic, 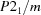

*a* = 6.7876 (7) Å
*b* = 14.2860 (14) Å
*c* = 7.8893 (8) Å
β = 113.562 (2)°
*V* = 701.23 (12) Å^3^
*Z* = 2
Mo *K*α radiation
μ = 21.39 mm^−1^
*T* = 200 K
0.28 × 0.14 × 0.13 mm

#### Data collection

Bruker SMART 1000 CCD diffractometer
Absorption correction: multi-scan (*SADABS*; Bruker, 2001 ▶) *T*_min_ = 0.459, *T*_max_ = 1.000
4257 measured reflections
1400 independent reflections
1176 reflections with *I* > 2σ(*I*)
*R*_int_ = 0.034

#### Refinement

*R*[*F* ^2^ > 2σ(*F* ^2^)] = 0.028
*wR*(*F* ^2^) = 0.075
*S* = 1.04
1400 reflections
88 parameters
H-atom parameters constrained
Δρ_max_ = 1.89 e Å^−3^
Δρ_min_ = −1.62 e Å^−3^

### 

Data collection: *SMART* (Bruker, 2007 ▶); cell refinement: *SAINT* (Bruker, 2007 ▶); data reduction: *SAINT*; program(s) used to solve structure: *SHELXS97* (Sheldrick, 2008 ▶); program(s) used to refine structure: *SHELXL97* (Sheldrick, 2008 ▶); molecular graphics: *ORTEP-3* (Farrugia, 1997 ▶) and *PLATON* (Spek, 2009 ▶); software used to prepare material for publication: *SHELXL97*.

## Supplementary Material

Crystal structure: contains datablock(s) global. DOI: 10.1107/S160053681203379X/hy2577sup1.cif

Additional supplementary materials:  crystallographic information; 3D view; checkCIF report

## Figures and Tables

**Table 1:** Hydrogen-bond geometry (Å, °)

*D*—H⋯*A*	*D*—H	H⋯*A*	*D*⋯*A*	*D*—H⋯*A*
N2—H2*N*⋯Br2^i^	0.92	2.79	3.665 (8)	161
C3—H3⋯Br1^ii^	0.95	2.90	3.689 (7)	141

Symmetry codes: (i) 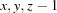; (ii) 
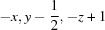
.

**Fig. 1.**
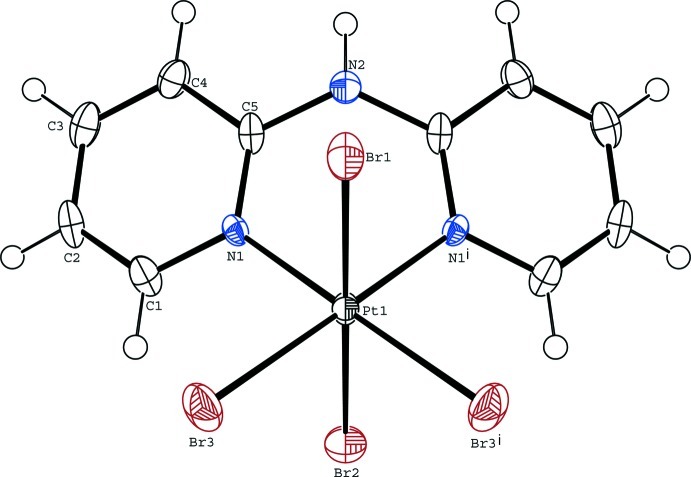
The molecular structure of the title complex. Displacement ellipsoids are drawn at the 50% probability level. [Symmetry code: (i) x, 1/2-y, z.]

**Fig. 2.**
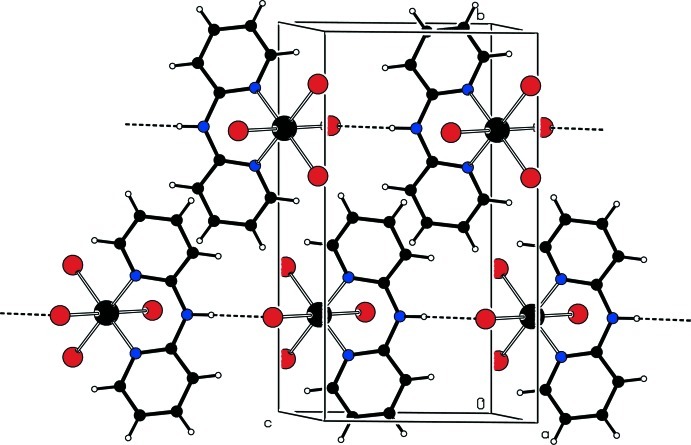
A view of the crystal packing of the title complex. Intermolecular N—H···Br hydrogen bonds are shown as dashed lines.

## supplementary crystallographic information

### Comment

The title complex, [PtBr_4_(dpa)] (dpa = di-2-pyridylamine, C_10_H_9_N_3_), is a structural isomer of the previously reported chlorido Pt^IV^ complex [PtCl_4_(dpa)] (Ha, 2011).

The Pt^IV^ ion is six-coordinated in a slightly distorted octahedral environment defined by two pyridine N atoms from a chelating dpa ligand and four Br^-^ anions (Fig. 1). The complex is disposed about a mirror plane, passing through the Pt1, Br1, Br2 and N2 atoms. The Pt—N and Pt—Br bond distances are comparable to those observed in the related Pt^II^ complex [PtBr_2_(dpa)] (Ha, 2012). In the crystal, the dpa ligand is not planar. The dihedral angle between the least-squares planes of the pyridine rings is 34.7 (2)°. The complex molecules are stacked in columns along the *a* axis and connected by intermolecular N—H···Br hydrogen bonds, forming chains along the *c* axis (Fig. 2, Table 1). Intermolecular π–π interactions between the pyridine rings are present, with a centroid–centroid distance of 3.667 (4) Å. Intermolecular C—H···Br hydrogen bonds are also observed (Table 1).

### Experimental

To a solution of K_2_PtCl_6_ (0.240 g, 0.49 mmol) and KBr (0.745 g, 6.26 mmol) in H_2_O (50 ml) was added di-2-pyridylamine (0.086 g, 0.50 mmol), and the mixture was stirred for 24 h at room temperature. The formed precipitate was separated by filtration, washed with H_2_O and acetone, and recrystallized from a mixture of *N,N*-dimethylformamide and ether to give a red powder (0.144 g). Crystals suitable for X-ray analysis were obtained by slow evaporation from a CH_3_CN solution at room temperature.

### Refinement

C-bound H atoms were positioned geometrically and allowed to ride on their respective parent atoms, with C—H = 0.95 Å and with *U*_iso_(H) = 1.2*U*_eq_(C). N-bound H atom was located from a difference Fourier map and then allowed to ride on its parent atom in the final cycles of refinement, with N—H = 0.92 Å and *U*_iso_(H) = 1.2*U*_eq_(N). The highest peak (1.89 e Å^-3^) and the deepest hole (-1.62 e Å^-3^) in the difference Fourier map are located 0.85 Å and 0.67 Å from atoms Pt1 and Br1, respectively.

### Crystal data

**Table:**

[PtBr_4_(C_10_H_9_N_3_)]	*F*(000) = 616
*M**_r_* = 685.93	*D*_x_ = 3.249 Mg m^−^^3^
Monoclinic, *P*2_1_/*m*	Mo *K*α radiation, λ = 0.71073 Å
Hall symbol: -P 2yb	Cell parameters from 2726 reflections
*a* = 6.7876 (7) Å	θ = 2.8–26.0°
*b* = 14.2860 (14) Å	µ = 21.39 mm^−^^1^
*c* = 7.8893 (8) Å	*T* = 200 K
β = 113.562 (2)°	Block, red
*V* = 701.23 (12) Å^3^	0.28 × 0.14 × 0.13 mm
*Z* = 2	

### Data collection

**Table:**

Bruker SMART 1000 CCD diffractometer	1400 independent reflections
Radiation source: fine-focus sealed tube	1176 reflections with *I* > 2σ(*I*)
Graphite monochromator	*R*_int_ = 0.034
φ and ω scans	θ_max_ = 26.0°, θ_min_ = 2.8°
Absorption correction: multi-scan (*SADABS*; Bruker, 2001)	*h* = −8→8
*T*_min_ = 0.459, *T*_max_ = 1.000	*k* = −17→15
4257 measured reflections	*l* = −9→9

### Refinement

**Table:**

Refinement on *F*^2^	Primary atom site location: structure-invariant direct methods
Least-squares matrix: full	Secondary atom site location: difference Fourier map
*R*[*F*^2^ > 2σ(*F*^2^)] = 0.028	Hydrogen site location: inferred from neighbouring sites
*wR*(*F*^2^) = 0.075	H-atom parameters constrained
*S* = 1.04	*w* = 1/[σ^2^(*F*_o_^2^) + (0.0388*P*)^2^ + 0.9524*P*] where *P* = (*F*_o_^2^ + 2*F*_c_^2^)/3
1400 reflections	(Δ/σ)_max_ < 0.001
88 parameters	Δρ_max_ = 1.89 e Å^−^^3^
0 restraints	Δρ_min_ = −1.62 e Å^−^^3^

### Special details

**Table:**

Geometry. All e.s.d.'s (except the e.s.d. in the dihedral angle between two l.s. planes) are estimated using the full covariance matrix. The cell e.s.d.'s are taken into account individually in the estimation of e.s.d.'s in distances, angles and torsion angles; correlations between e.s.d.'s in cell parameters are only used when they are defined by crystal symmetry. An approximate (isotropic) treatment of cell e.s.d.'s is used for estimating e.s.d.'s involving l.s. planes.
Refinement. Refinement of *F*^2^ against ALL reflections. The weighted *R*-factor *wR* and goodness of fit *S* are based on *F*^2^, conventional *R*-factors *R* are based on *F*, with *F* set to zero for negative *F*^2^. The threshold expression of *F*^2^ > σ(*F*^2^) is used only for calculating *R*-factors(gt) *etc*. and is not relevant to the choice of reflections for refinement. *R*-factors based on *F*^2^ are statistically about twice as large as those based on *F*, and *R*- factors based on ALL data will be even larger.

### Fractional atomic coordinates and isotropic or equivalent isotropic displacement parameters (Å^2^)

**Table:**

	*x*	*y*	*z*	*U*_iso_*/*U*_eq_	
Pt1	0.02241 (6)	0.2500	0.81398 (5)	0.01565 (14)	
Br1	−0.30563 (16)	0.2500	0.52614 (15)	0.0260 (3)	
Br2	0.34926 (18)	0.2500	1.10292 (15)	0.0298 (3)	
Br3	−0.14760 (13)	0.13183 (5)	0.93509 (11)	0.0306 (2)	
N1	0.1651 (8)	0.1500 (3)	0.7074 (8)	0.0145 (11)	
N2	0.1284 (13)	0.2500	0.4576 (11)	0.0193 (17)	
H2N	0.1473	0.2500	0.3485	0.023*	
C1	0.2280 (12)	0.0668 (5)	0.7949 (11)	0.0251 (16)	
H1	0.2303	0.0591	0.9153	0.030*	
C2	0.2881 (12)	−0.0059 (4)	0.7148 (11)	0.0250 (16)	
H2	0.3358	−0.0632	0.7797	0.030*	
C3	0.2786 (11)	0.0046 (5)	0.5396 (11)	0.0257 (17)	
H3	0.3095	−0.0471	0.4784	0.031*	
C4	0.2249 (11)	0.0890 (5)	0.4520 (10)	0.0224 (15)	
H4	0.2227	0.0972	0.3318	0.027*	
C5	0.1730 (11)	0.1634 (4)	0.5426 (10)	0.0193 (15)	

### Atomic displacement parameters (Å^2^)

**Table:**

	*U*^11^	*U*^22^	*U*^33^	*U*^12^	*U*^13^	*U*^23^
Pt1	0.0193 (2)	0.0115 (2)	0.0184 (2)	0.000	0.00991 (17)	0.000
Br1	0.0226 (6)	0.0189 (5)	0.0313 (6)	0.000	0.0053 (5)	0.000
Br2	0.0315 (6)	0.0315 (6)	0.0222 (6)	0.000	0.0063 (5)	0.000
Br3	0.0381 (5)	0.0239 (4)	0.0393 (5)	−0.0026 (3)	0.0254 (4)	0.0072 (3)
N1	0.014 (3)	0.012 (2)	0.017 (3)	−0.002 (2)	0.005 (2)	0.003 (2)
N2	0.023 (5)	0.017 (4)	0.019 (4)	0.000	0.010 (4)	0.000
C1	0.029 (4)	0.019 (3)	0.030 (4)	0.002 (3)	0.014 (3)	0.007 (3)
C2	0.023 (4)	0.012 (3)	0.042 (5)	0.001 (3)	0.015 (4)	0.006 (3)
C3	0.022 (4)	0.018 (4)	0.041 (5)	−0.002 (3)	0.017 (4)	−0.008 (3)
C4	0.023 (4)	0.019 (3)	0.028 (4)	0.001 (3)	0.014 (3)	−0.005 (3)
C5	0.018 (4)	0.011 (3)	0.031 (4)	0.000 (3)	0.012 (3)	0.001 (3)

### Geometric parameters (Å, º)

**Table:**

Pt1—N1	2.082 (5)	C1—C2	1.360 (10)
Pt1—Br3	2.4446 (7)	C1—H1	0.9500
Pt1—Br1	2.4642 (12)	C2—C3	1.366 (11)
Pt1—Br2	2.4647 (12)	C2—H2	0.9500
N1—C5	1.337 (9)	C3—C4	1.366 (10)
N1—C1	1.356 (8)	C3—H3	0.9500
N2—C5^i^	1.382 (7)	C4—C5	1.401 (9)
N2—C5	1.382 (7)	C4—H4	0.9500
N2—H2N	0.9200		
			
N1^i^—Pt1—N1	86.6 (3)	C5^i^—N2—C5	127.1 (8)
N1^i^—Pt1—Br3^i^	93.02 (13)	C5^i^—N2—H2N	111.6
N1—Pt1—Br3^i^	179.26 (15)	C5—N2—H2N	111.6
N1^i^—Pt1—Br3	179.26 (15)	N1—C1—C2	121.7 (7)
N1—Pt1—Br3	93.02 (13)	N1—C1—H1	119.1
Br3^i^—Pt1—Br3	87.35 (4)	C2—C1—H1	119.1
N1^i^—Pt1—Br1	91.29 (16)	C1—C2—C3	118.9 (7)
N1—Pt1—Br1	91.29 (16)	C1—C2—H2	120.5
Br3^i^—Pt1—Br1	88.07 (3)	C3—C2—H2	120.5
Br3—Pt1—Br1	88.07 (3)	C4—C3—C2	120.3 (6)
N1^i^—Pt1—Br2	88.95 (15)	C4—C3—H3	119.9
N1—Pt1—Br2	88.95 (16)	C2—C3—H3	119.9
Br3^i^—Pt1—Br2	91.69 (3)	C3—C4—C5	118.8 (7)
Br3—Pt1—Br2	91.69 (3)	C3—C4—H4	120.6
Br1—Pt1—Br2	179.67 (3)	C5—C4—H4	120.6
C5—N1—C1	119.6 (6)	N1—C5—N2	120.9 (6)
C5—N1—Pt1	120.1 (4)	N1—C5—C4	120.3 (6)
C1—N1—Pt1	119.9 (4)	N2—C5—C4	118.9 (6)
			
N1^i^—Pt1—N1—C5	−39.4 (6)	C1—C2—C3—C4	4.8 (11)
Br3—Pt1—N1—C5	139.9 (5)	C2—C3—C4—C5	−2.1 (10)
Br1—Pt1—N1—C5	51.8 (5)	C1—N1—C5—N2	−174.2 (7)
Br2—Pt1—N1—C5	−128.4 (5)	Pt1—N1—C5—N2	13.3 (9)
N1^i^—Pt1—N1—C1	148.0 (4)	C1—N1—C5—C4	6.5 (10)
Br3—Pt1—N1—C1	−32.6 (5)	Pt1—N1—C5—C4	−166.1 (5)
Br1—Pt1—N1—C1	−120.7 (5)	C5^i^—N2—C5—N1	34.0 (13)
Br2—Pt1—N1—C1	59.0 (5)	C5^i^—N2—C5—C4	−146.7 (7)
C5—N1—C1—C2	−3.8 (10)	C3—C4—C5—N1	−3.6 (10)
Pt1—N1—C1—C2	168.8 (6)	C3—C4—C5—N2	177.0 (7)
N1—C1—C2—C3	−1.9 (11)		

Symmetry code: (i) *x*, −*y*+1/2, *z*.

### Hydrogen-bond geometry (Å, º)

**Table:**

*D*—H···*A*	*D*—H	H···*A*	*D*···*A*	*D*—H···*A*
N2—H2*N*···Br2^ii^	0.92	2.79	3.665 (8)	161
C3—H3···Br1^iii^	0.95	2.90	3.689 (7)	141

Symmetry codes: (ii) *x*, *y*, *z*−1; (iii) −*x*, *y*−1/2, −*z*+1.
